# Lifestyle habits and gastric cancer in an East Asian population: a Mendelian randomization study

**DOI:** 10.3389/fonc.2023.1224753

**Published:** 2023-09-04

**Authors:** Yuegui Tan, Zhao Wei, Kun Liu, Yuzhen Qin, Wenqi Hui

**Affiliations:** ^1^ Department of Pharmacy, Xi’an Fifth Hospital, Xian, Shaanxi, China; ^2^ Department of Medicinal Chemistry, School of Pharmacy, Air Force Medical University, Xi’an, Shaanxi, China; ^3^ Department of Epidemiology, Ministry of Education Key Lab of Hazard Assessment and Control in Special Operational Environment, School of Public Health, Air Force Medical University, Xi’an, Shaanxi, China; ^4^ Xi’an Jiaotong-liverpool University, XJTLU Wisdom Lake Academy of Pharmacy, Xian, Shaanxi, China

**Keywords:** gastric cancer, Mendelian randomization, smoking, alcohol consumption, tea intake, coffee intake

## Abstract

**Background:**

Epidemiological evidence suggests an association between lifestyle habits (smoking, alcohol consumption, tea, coffee intake, etc.) and gastric cancer (GC). However, the causal relationship remains uncertain. Therefore, the purpose of this study was to ascertain whether there is a causal connection between them.

**Methods:**

Two-sample Mendelian randomization (MR) analysis was performed using the publicly available Genome Wide Association Study summary datasets using six methods: inverse variance weighting (IVW), weighted median, MR using a Robust Adjusted Profile Score (MR.Raps), MR using a Robust Adjusted Profile Score (MR-PRESSO), Radial regression of MR, and Causal Analysis Using Summary Effect Estimates (CAUSE). A sensitivity analysis was conducted to assess the robustness of the results.

**Results:**

In an East Asian population, we found that increased tea intake reduced the risk of GC [odds ratio (OR)= 0.90, 95% confidence interval (CI)= 0.82-0.99, P = 0.037] while there was a positive association between smoking and GC (OR = 1.58, 95% CI = 1.04-2.39, P = 0.032). No causal relationship between alcohol and coffee intake and GC. Sensitivity analyses demonstrated the robustness of these causal associations.

**Conclusions:**

Our study suggests that tea intake may reduce the risk of GC, for which smoking is a potential risk factor. Nevertheless, a larger and more diverse sample size is needed for further validation.

## Introduction

According to the International Agency for Research on Cancer’s (IARC) 2020 findings, gastric cancer (GC), a highly aggressive and lethal malignancy, is the fourth most prevalent cause of cancer mortality globally and the fifth most common cancer overall (1–3). According to statistics (3), there were 479,000 new cases of gastric cancer and 374,000 deaths in China in 2020, accounting for 44.0% and 48.6% of new gastric cancer cases and deaths worldwide, respectively. The prevalence of cancer screening and improvements in food hygiene have led to a gradual decline in the incidence and mortality of GC over the past half-century. However, in recent years, the incidence of GC has rebounded in some countries due to the disruption of the gastric microbiological environment as a result of the inappropriate use of antibiotics and acid inhibitors (4, 5). Due to the lack of clinical signs and symptoms of early GC, most patients are in the middle or late stages at the time of diagnosis. The high cost of clinical treatment, poor prognosis, and short survival period add to the disease burden of GC. Therefore, the study of its risk factors is crucial for the prevention and treatment of gastric cancer.

Currently, various factors have been reported that may correlate with the development of GC. In addition to inflammation (6), infection (7), environmental, immune, and genetic factors (8), lifestyle factors may also influence lifetime cancer risk (9). In observational studies, smoking (10), alcohol consumption (11), coffee (12), and tea intake (13, 14) have been identified as factors associated with GC. Nevertheless, as a result of inconsistent findings and potential biases (15, 16), including residual confounding, reverse causality, and misclassification, it remains unclear whether these associations between lifestyle habits are causal.

Mendel’s second law states that genotypes are fixed before illness onset and alleles are randomly assigned to progeny gametes at the time of gamete production. In addition, as a result of genetic variation being used as an exposure tool (e.g., smoking, alcohol consumption, coffee, and tea intake), Mendelian randomization (MR) studies can improve causal inference by reducing residual confounding and reverse causality (17–19). Here, we conducted an MR study to explore genetically predicted associations between smoking, alcohol, coffee, and tea intake with gastric cancer-related phenotypes.

## Methods

### Data sources

We downloaded publicly available abstract-level genome-wide association study (GWAS) datasets from the MRC Integrative Epidemiology Unit (IEU) at the University of Bristol (https://gwas.mrcieu.ac.uk/). We conducted two-sample bivariate MR analyses to assess the causal effects of lifestyle factors (smoking, alcohol, coffee, and tea intake) and GC.

We selected relevant phenotypes (smoking, alcohol, coffee, and tea intake) from European and East Asian populations as exposures to analyze their correlation with GC in East Asian populations, and thus distinguish whether this correlation is biased by population stratification. The genome-wide Association Study (GWAS) pooled statistics for GC included 34,652 subjects of East Asian descent (6,563 GC cases and 195,745 controls) (20). **Table S1** shows detailed information on the data used. There were no raw data requirements for this MR study since we used publicly available or published data. Ethical approval for all GWAS studies was obtained from the appropriate institutional review board. Therefore, ethics committee approval is no longer required for this study.

### Mendelian randomization design and instrumental variables

When evaluating the causal connection between an exposure and an outcome in MR, genetic variant(s) are employed as IVs. The fundamental conditions for a genetic variant to satisfy an IV are summarized as follows: (1) the variation and exposure are connected; (2) the variation has no connection to any confounding factor in the exposure-outcome relationship; (3) with the possible exception of its relationship to the exposure, the variation has no effect on the result.

At the outset of the research design, single nucleotide polymorphisms (SNPs) (*P* < 5 × 10^-5^) that are strongly related with exposure were selected as IVs. We only used independent SNPs with r^2^ < 0.01 within a distance of 5,000 kb in order to remove the chain disequilibrium (LD) bias (21). Therefore, the default parameters of the TwoSampleMR package in R (physical distance threshold, 5,000 kb, r^2^ < 0.01) were set for aggregating the data and clipping possible LD genetic variation. Furthermore, we used the PhenoSCanner database, which includes a wealth of information on SNPs that may be associated with disease and phenotype (22), to exclude SNPs that were associated between lifestyle factors and GC. Finally, our analysis for palindromic SNPs was excluded those whose minor allele frequency (MAF) was less than 0.3 (23). In order to prevent weak instrumental variable bias, we calculated the F-statistics for each IV-SNP according to this formula: F= (β/SE)^2^ (24). IVs that had an F-statistic of less than 10 were regarded as weak instruments and were rejected.

### Mendelian randomization analyses

Inverse variance weighted (IVW) analysis was employed as the primary approach after choosing the suitable SNPs for exposures, and we augmented this with the use of numerous MR techniques based on various IVs assumptions, including weighted median (WM) (25), MR using a Robust Adjusted Profile Score (MR.Raps) (26), MR-Pleiotropy Residual Sum and Outlier (MR-PRESSO) (27), Radial regression of MR (Radial MR) (28) and causal analysis using summary effects estimation (CAUSE) (29) as sensitivity analysis to validate the robustness of the main IVW estimates. IVW can force the intercept term in weighted linear regression to zero to determine the causal effect value, and all three assumptions must be met in order for the causal effect estimation to be unbiased. IVW can be used to integrate multiple SNPs to obtain consistent estimates of causal effect values (30). For WM, at least 50% of SNPs are effective as IVs, which can generate strong causal effect values (31). The MR-PRESSO method can be used to identify anomalous data points and generate revised estimates after deletion (32). MR-RAPS can make robust inferences when containing weak IVs (33). Radial MR identifies outliers and repeats the MR analysis after eliminating heterogeneous SNPs (28).

To improve the accuracy of causal effects, we additionally calculated Cochran’s Q statistic to quantify heterogeneity across causal estimates of all SNPs in the IVW technique. In order to evaluate the horizontal pleiotropy impact of IVs, MR-Egger regression was performed. MR-Egger can use the intercept term to evaluate pleiotropy. When the intercept term is equal to zero, the results of MR-Egger are consistent with IVW, proving the absence of horizontal pleiotropy (34). For selected IVs, we used the MR-PRESSO global test to examine whether there were any outliers with variant-specific causal estimates that were significantly different from the rest. Using the leave-one-plot function from the TwoSampleMR package in R, we carried out a leave-one-out analysis to further confirm the stability of the analytic results (24).

### Statistical analysis

MR analysis stastistics were performed using the following R version 4.2.0 packages: “TwoSampleMR” (24, 35), “MR-PRESSO” (36), and “CAUSE” (37).

## Results

### Instrumental variables

To prepare our analyses for investigating the effect of lifestyle habits on GC risk, we performed chain imbalance clustering, and palindromic SNPs. We additionally exclude SNPs related to confounding factors by searching for pleiotropic associations between the SNPs used and other traits in PhenoScanner (www.phenoscanner.medschl.cam.ac.uk), and did not find any such associations. Thereafter, 135, 66, 50, and 56 SNPs from East Asian populations and 86, 179, 119, and 151 SNPs from European populations were included as IVs for smoking, alcohol intake, coffee intake, and tea intake, respectively. The F-statistic indicated no bias due to weak instrumentation (F > 10, **Table S2-9**).

### Effect of lifestyle factors in Europeans on GC

**Figure 1:**
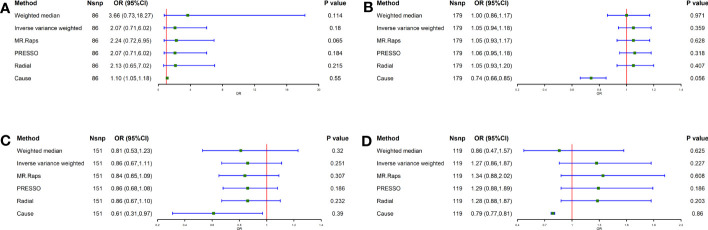
Causal effect of Lifestyle Factors in European on GC. **(A)** Smoking and GC; **(B)** Alcohol intake and GC; **(C)** Tea intake and GC; **(D)** Coffee intake and GC; The green point means the effect (OR). GC, Gastric cancer.

As shown in **Figure 1**, the IVW analysis showed no causal relationship between smoking [Odds ratio (OR) = 2.07, 95% confidence interval (CI) = 0.71-6.02, *P* = 0.180], alcohol consumption (OR = 1.05, 95% CI = 0.94-1.18, *P* = 0.359), coffee intake (OR = 1.27, 95% CI = 0.86-1.87, *P* = 0.227) and tea intake (OR = 0.86, 95% CI = 0.67-1.11, *P* = 0.251) and GC. There was no evidence of substantial heterogeneity (*P_Q_* > 0.05 in Cochran’s Q test and *P*_intercept_> 0.05 in MR-Egger) or horizontal pleiotropy (*P*_global_ > 0.05 in MR-PRESSO global test) in these causal estimates when sensitivity analyses were performed (**Table S10**). The results of the analysis of scatterplots, forest plots, funnel plots are shown in **Supplementary Figures S1**–**S3**. Meanwhile, this causal effect cannot be attributed to any single IV, as indicated by the leave-one-out analysis (**Supplementary Figure S4**).

### Effect of lifestyle factors in Eastern Asians on GC

**Figure 2:**
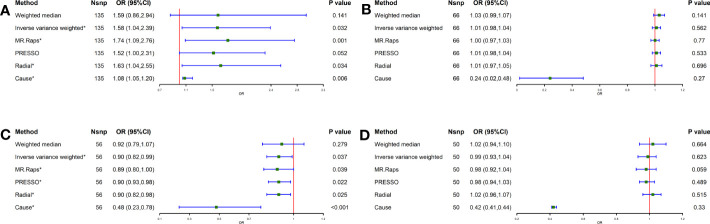
Causal effect of Lifestyle Factors in Eastern Asia on GC. **(A)** Smoking and GC; **(B)** Alcohol intake and GC; **(C)** Tea intake and GC; **(D)** Coffee intake and GC; The green point means the effect (OR). GC, Gastric cancer.

To determine the correlation between lifestyle habits and GC, the IVW method showed a possible positive association between smoking and GC (OR = 1.58, 95% CI = 1.04-2.39, *P* = 0.032) and a negative association between tea intake and GC (OR = 0.90, 95% CI = 0.82-0.99, *P* = 0.037) (**Figure 2**). In addition, for alcohol consumption and coffee intake, no causal relationship was found between either and GC (alcohol consumption: *P* = 0.562, coffee intake: *P* = 0.623) (**Figure 2**). Furthermore, the MR-Egger and MR-PRESSO methodologies, as well as Cochran’s Q-statistic, show that there is no heterogeneity or pleiotropy in these studies (**Table S11**). The results of the scatterplot, forest plot, and funnel plot analyses are shown in **Supplementary Figures S5**–**7**, respectively. All figures are consistent with the results of the above analyses. No one SNP was found to have a significant impact on the overall outcome of GC for these causal associations, according to the leave-one-out sensitivity analysis (**Supplementary Figure S8**).

## Discussion

Our study examined the causal relationship between lifestyle factors (smoking, alcohol, coffee, and tea intake) and GC lesions using a two-sample MR approach. We come to the conclusion that in an East Asian community, tea consumption is adversely linked with GC whereas smoking is favorably associated with increasing incidence of GC. We also found no causal relationship between alcohol and coffee intake with GC. Also, certain lifestyle choices (smoking, alcohol, tea, and coffee intake) as exposure in the European population and outcome analysis in the East Asian population did not reveal a causal relationship. Our findings were valid and stable in IVW analyses before and after excluding outlier SNPs and were also stable in sensitivity analyses.

Among the various habits that play a role in GC development, the impact of smoking has been considered. Numerous studies have shown that smokers are more likely than non-smokers to get stomach cancer. The study showed that never smoking or quitting for more than 10 years ago were associated with a reduced risk of GC at a mean follow-up of 11.4 years (OR = 0.64, 95% CI = 0.54-0.75) (38). Moy et al. found an estimated risk of 80% for GC in a group of smokers (39). Smoking was named by the IARC as a risk factor for the development of GC in 2002, and it was also noted that there is enough evidence to imply a causal link between smoking and GC (40). Tobacco is a biologically plausible tumour-promoting behavior and cigarette smoke has been reported to contain more than 7,000 toxic chemicals, including carcinogens such as nitrosamines, polycyclic aromatic hydrocarbons, acrylamides, volatile organic compounds and cadmium, which can increase the risk of cancer (10, 41). There is a possibility that these carcinogens and toxins may directly damage DNA. Since DNA controls the growth and function of cells, damages to DNA can alter normal cell growth patterns and further cause abnormal gastric epithelial cells to become cancerous (42, 43). Secondly, the bacterium *Helicobacter pylori* (*H. pylori*) is a significant risk factor for GC and its complete eradication has been shown to slow the occurrence of GC (44), while Pan et al. also suggest that smoking is not only a known risk factor for the development of GC, but is also linked to the failure of *H. pylori* eradication (38). What’s more, tobacco smoke reduces gastric defence mechanisms (45) and levels in mucus (46) gastric secretions by decreasing vitamin C concentrations and also increases bile reflux (47). Stomach cancer precursor lesions may form and grow as a result of increased bile acid concentrations in stomach contents and compromised gastric defense systems (48).

Our findings regarding the negative association between tea intake and GC are consistent with most, but not all, previous studies. The inverse association between tea consumption and GC can be explained by a number of biological processes. In previous studies, polyphenols in tea have been hypothesized to prevent cancer by regulating DNA methylation, histone modifications, and epigenetic abnormalities in microRNA (49). Studies has also revealed that the major polyphenols in tea (theaflavins and catechins) have inhibitory effects on cancer cell proliferation, tumor growth, angiogenesis, metastasis and inflammation while also inducing apoptosis to prevent tumourigenesis (50, 51). Additionally, several polyphenols in tea, such as theobromine and theaflavin, have been shown to have antioxidant properties in laboratory studies (52).

Our results for alcohol intake and coffee intake did not indicate a causal relationship with GC, possibly due to the following reasons: GC in cardia and non-cardia sites may have different etiologies. As for the anatomic site of gastric cancer, previous studies have evaluated the association between alcohol intake and site-specific risk, but the results have been inconsistent (53, 54). Furthermore, in a prospective study of Japanese men and women, light to heavy alcohol consumption was found to be associated with an increased risk of gastric cancer incidence in a dose-response manner in men, whereas a null association was seen in women (55). Meanwhile, regarding the subsites of GC, a significant positive correlation between coffee consumption and gastric cardia cancer and zero correlation with gastric non-cardia cancer was observed in the NIH-AARP study (56). It is worth noting that in this study, we did not categorize gastric cancer and the gender of the population, etc., which may have made our findings show that there is no causal relationship between alcohol consumption and GC.

The greatest strength of this study is the two-sample MR study design. As a result, MR analyses is an excellent method for understanding whether exposure factors contribute to a disease’s development. Moreover, we investigated the possibility of pleiotropy by using the most recent methodological developments. Based on the results of different sensitivity analyses, little evidence of pleiotropy can be found. As a result, our causal estimates are reliable and robust.

It is important to note, however, that our MR analyses do have some limitations. Firstly, due to the limited information available in the IEU GWAS database, it was not possible to assess the effect of type of tea. Secondly, the identification and evaluation of abnormal genetic variants involves multiple methods, however mediators or pleiotropy cannot be completely excluded as possible influences. Thirdly, the intensity of IV depends on the sample size of the GWAS, and a larger GWAS is needed to identify more genetic variation in MR. Finally, our results are instructive for disease prevention, but further validation and analysis are still needed due to the limitations of the selected GAWS data.

## Conclusion

In summary, the results of the MR analysis suggest a causal relationship between smoking and tea intake with GC in the East Asian population. This result has some implications for the prevention and diagnosis of GC. However, due to the limitations of the study, this hypothesis needs to be supported by more high-quality cohort studies or randomized controlled trials with a larger and more diverse samples population.

## Data availability statement

The original contributions presented in the study are included in the article/**Supplementary Material**, further inquiries can be directed to the corresponding author.

## Author contributions

WH designed this study protocol and supervised the study. YG and ZW drafted the manuscript. KL performed the data analysis. YQ polished the manuscript. All authors contributed to the article and approved the submitted version.

## Supplementary material

Supplementary Figure 1 Scatterplot of the causal association between Lifestyle Factors in European and GC. **(A)** Smoking and GC; **(B)** Alcohol intake and GC; **(C)** Coffee intake and GC; **(D)** Tea intake and GC. GC, Gastric cancer.

Supplementary Figure 2 Forest plot of the causal association between Lifestyle Factors in European and GC. **(A)** Smoking and GC; **(B)** Alcohol intake and GC; **(C)** Coffee intake and GC; **(D)** Tea intake and GC. GC, Gastric cancer.

Supplementary Figure 3 Funnel plot of the causal association between Lifestyle Factors in European and GC. **(A)** Smoking and GC; **(B)** Alcohol intake and GC; **(C)** Coffee intake and GC; **(D)** Tea intake and GC. GC, Gastric cancer.

Supplementary Figure 4 Leave-one-out test plot of the causal association between Lifestyle Factors in European and GC. **(A)** Smoking and GC; **(B)** Alcohol intake and GC; **(C)** Coffee intake and GC; **(D)** Tea intake and GC. GC, Gastric cancer.

Supplementary Figure 5 Scatterplot of the causal association between Lifestyle Factors in Eastern Asia and GC. **(A)** Smoking and GC; **(B)** Alcohol intake and GC; **(C)** Coffee intake and GC; **(D)** Tea intake and GC. GC, Gastric cancer.

Supplementary Figure 6 Forest plot of the causal association between Lifestyle Factors in Eastern Asia and GC. **(A)** Smoking and GC; **(B)** Alcohol intake and GC; **(C)** Coffee intake and GC; **(D)** Tea intake and GC. GC, Gastric cancer.

Supplementary Figure 7 Funnel plot of the causal association between Lifestyle Factors in Eastern Asia and GC. **(A)** Smoking and GC; **(B)** Alcohol intake and GC; **(C)** Coffee intake and GC; **(D)** Tea intake and GC. GC, Gastric cancer.

Supplementary Figure 8 Leave-one-out test plot of the causal association between Lifestyle Factors in Eastern Asia and GC. **(A)** Smoking and GC; **(B)** Alcohol intake and GC; **(C)** Coffee intake and GC; **(D)** Tea intake and GC. GC, Gastric cancer.
