# Supplementary figures and images for: Lifestyle habits and gastric cancer in an East Asian population: a Mendelian randomization study
Source: Front Oncol. 2023 Sep 4;13:1224753. doi: 10.3389/fonc.2023.1224753 (PMC10507616; doi:10.3389/fonc.2023.1224753)

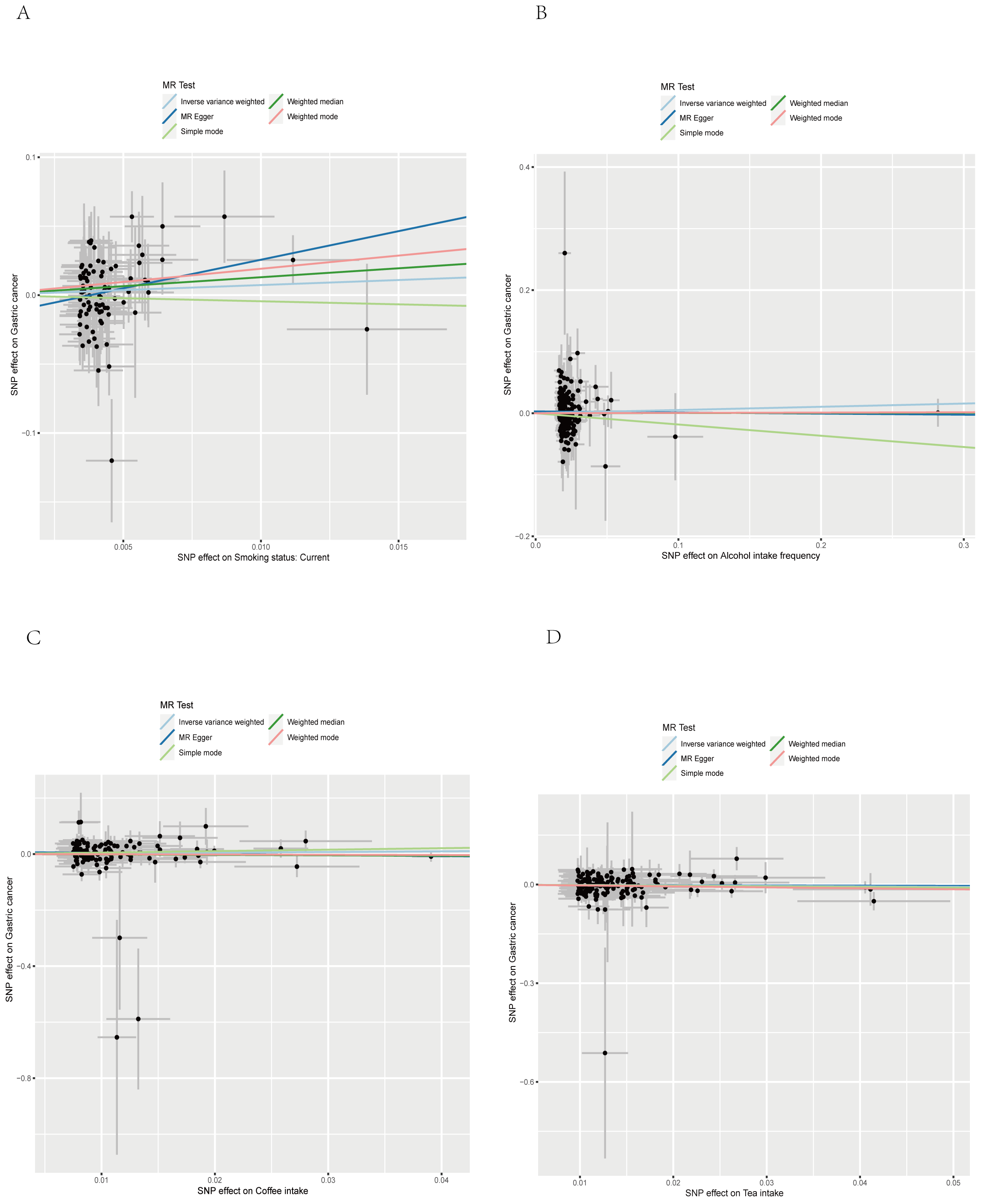

Supplement: Supplementary Figure 1 — Scatterplot of the causal association between Lifestyle Factors in European and GC. (A) Smoking and GC; (B) Alcohol intake and GC; (C) Coffee intake and GC; (D) Tea intake and GC. GC, Gastric cancer. [file Image_1.tif]

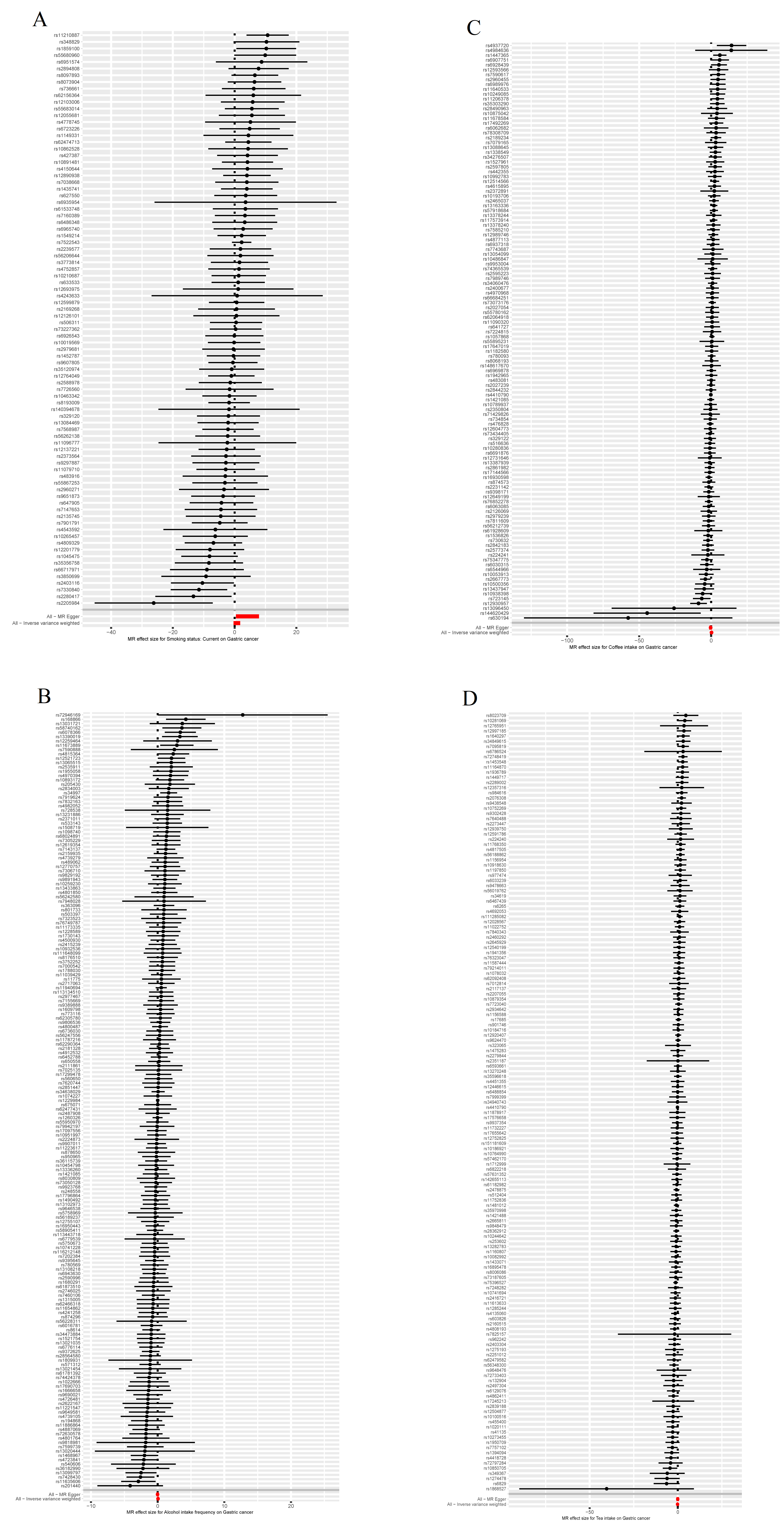

Supplement: Supplementary Figure 2 — Forest plot of the causal association between Lifestyle Factors in European and GC. (A) Smoking and GC; (B) Alcohol intake and GC; (C) Coffee intake and GC; (D) Tea intake and GC. GC, Gastric cancer. [file Image_2.tif]

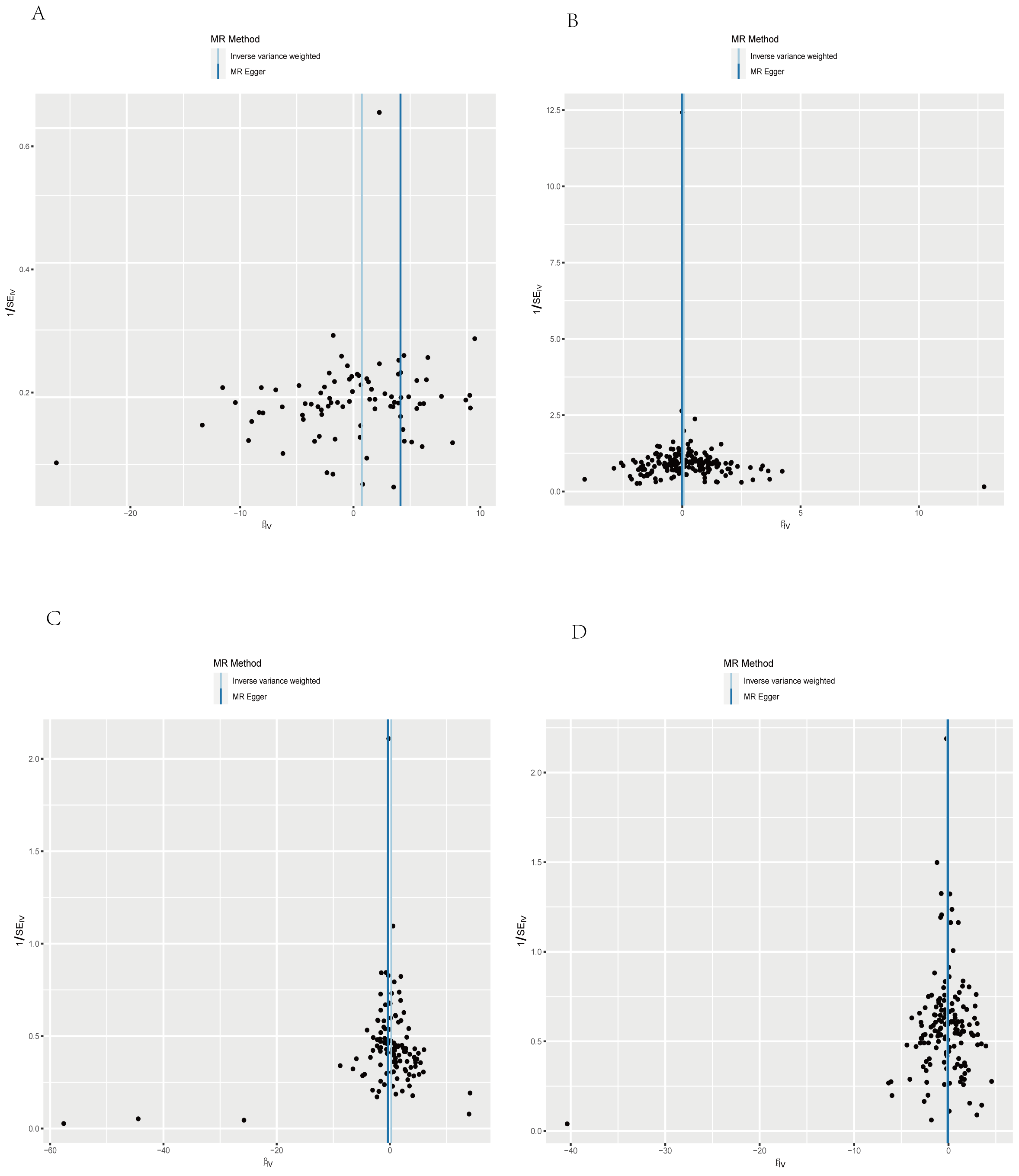

Supplement: Supplementary Figure 3 — Funnel plot of the causal association between Lifestyle Factors in European and GC. (A) Smoking and GC; (B) Alcohol intake and GC; (C) Coffee intake and GC; (D) Tea intake and GC. GC, Gastric cancer. [file Image_3.tif]

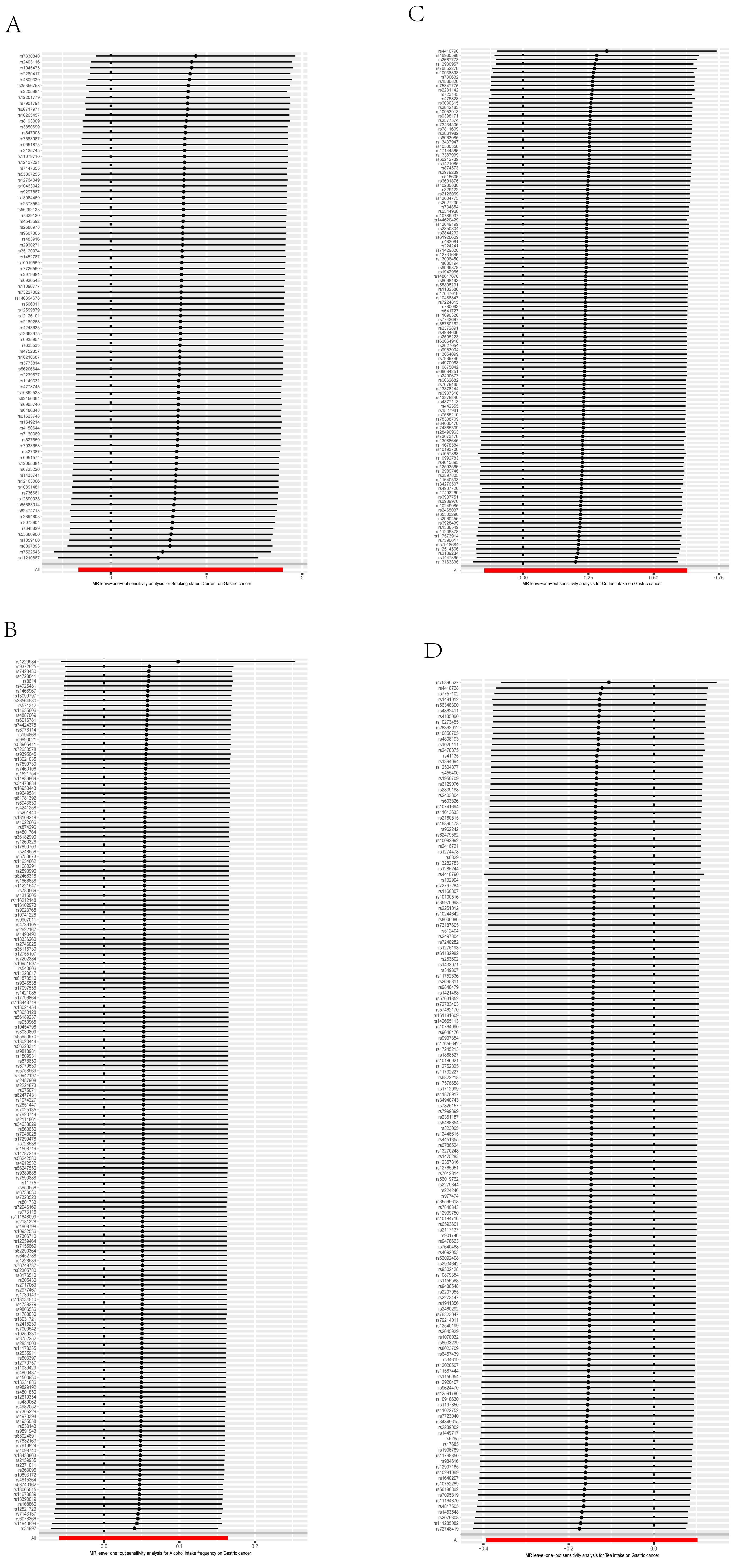

Supplement: Supplementary Figure 4 — Leave-one-out test plot of the causal association between Lifestyle Factors in European and GC. (A) Smoking and GC; (B) Alcohol intake and GC; (C) Coffee intake and GC; (D) Tea intake and GC. GC, Gastric cancer. [file Image_4.tif]

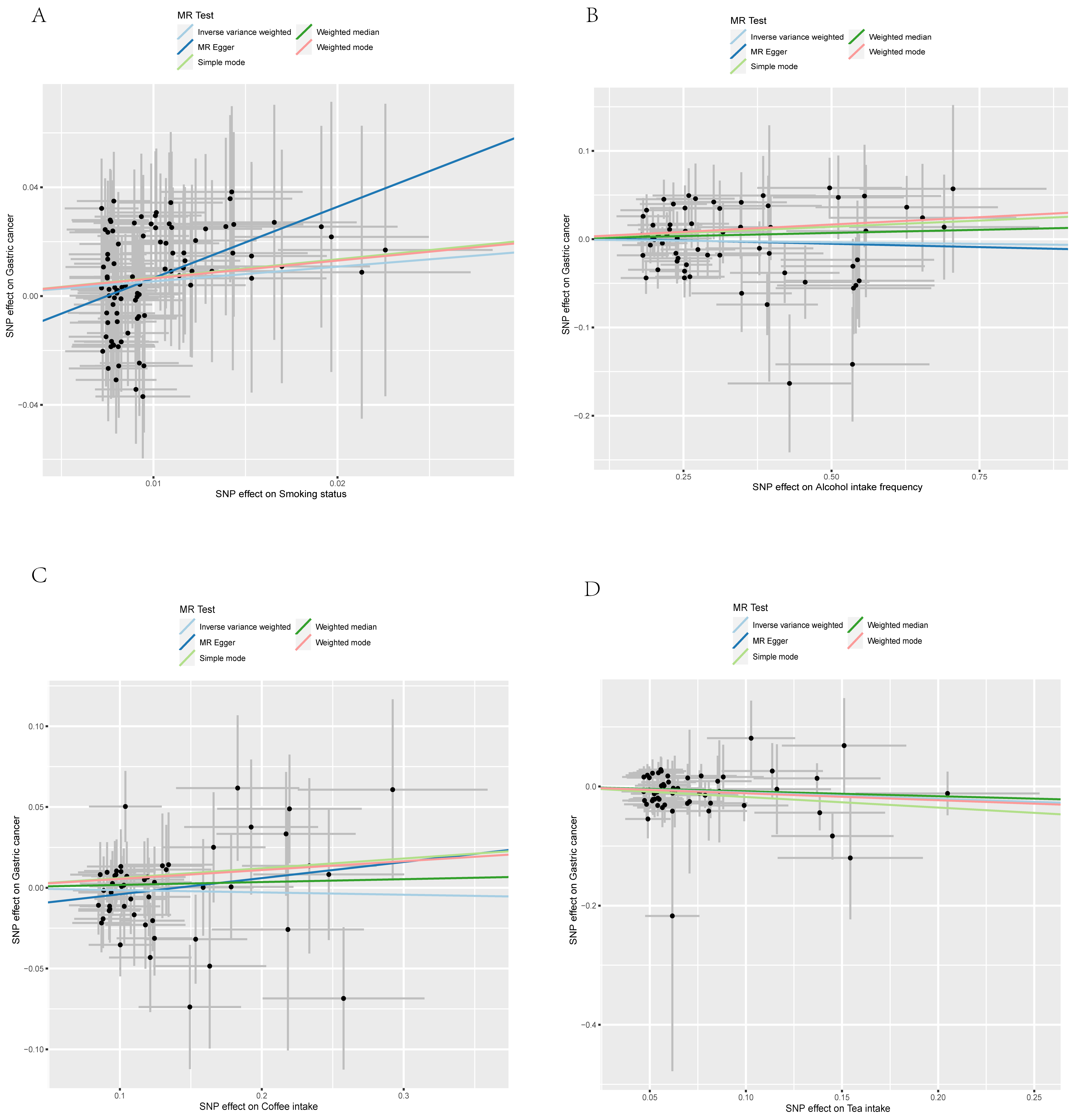

Supplement: Supplementary Figure 5 — Scatterplot of the causal association between Lifestyle Factors in Eastern Asia and GC. (A) Smoking and GC; (B) Alcohol intake and GC; (C) Coffee intake and GC; (D) Tea intake and GC. GC, Gastric cancer. [file Image_5.tif]

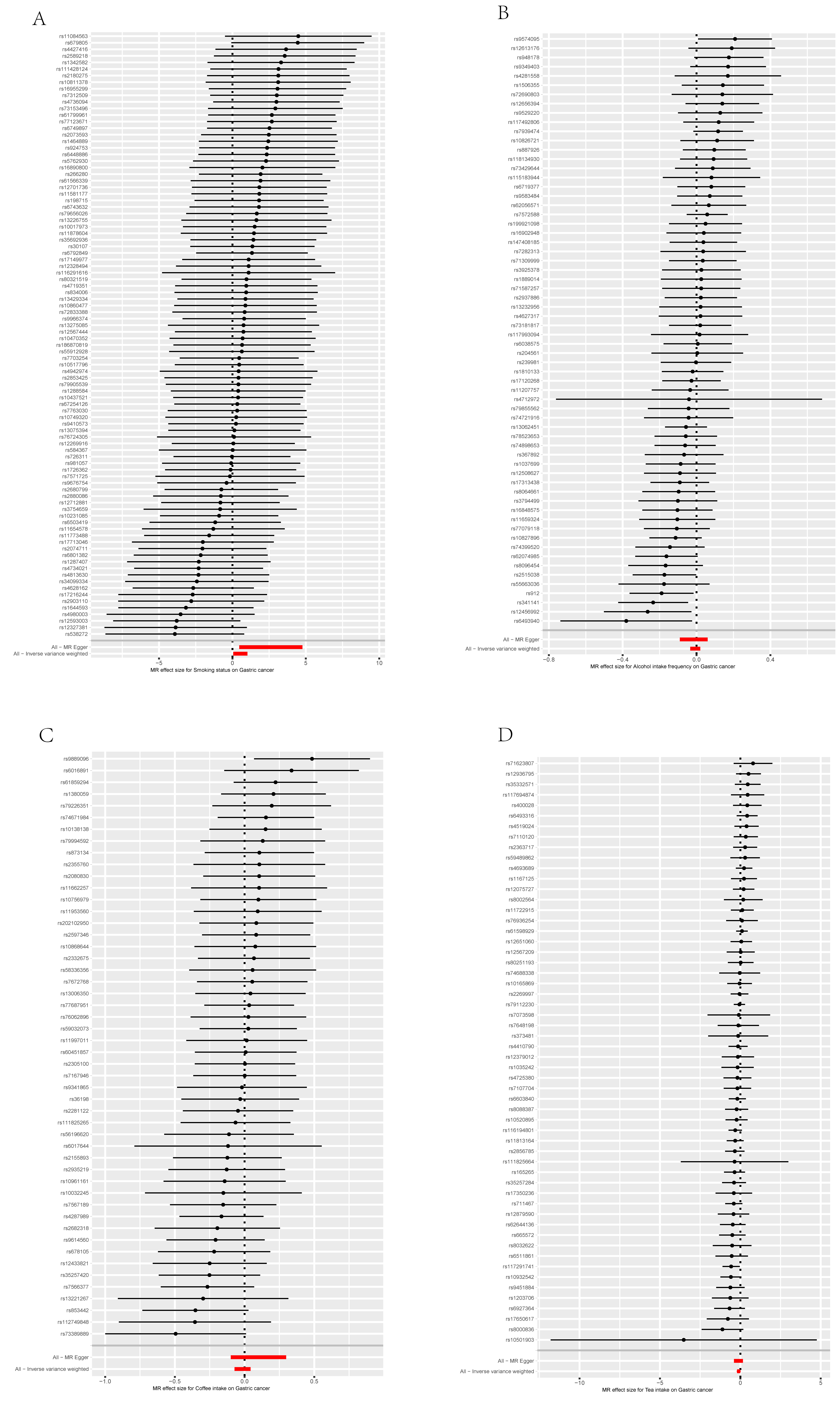

Supplement: Supplementary Figure 6 — Forest plot of the causal association between Lifestyle Factors in Eastern Asia and GC. (A) Smoking and GC; (B) Alcohol intake and GC; (C) Coffee intake and GC; (D) Tea intake and GC. GC, Gastric cancer. [file Image_6.tif]

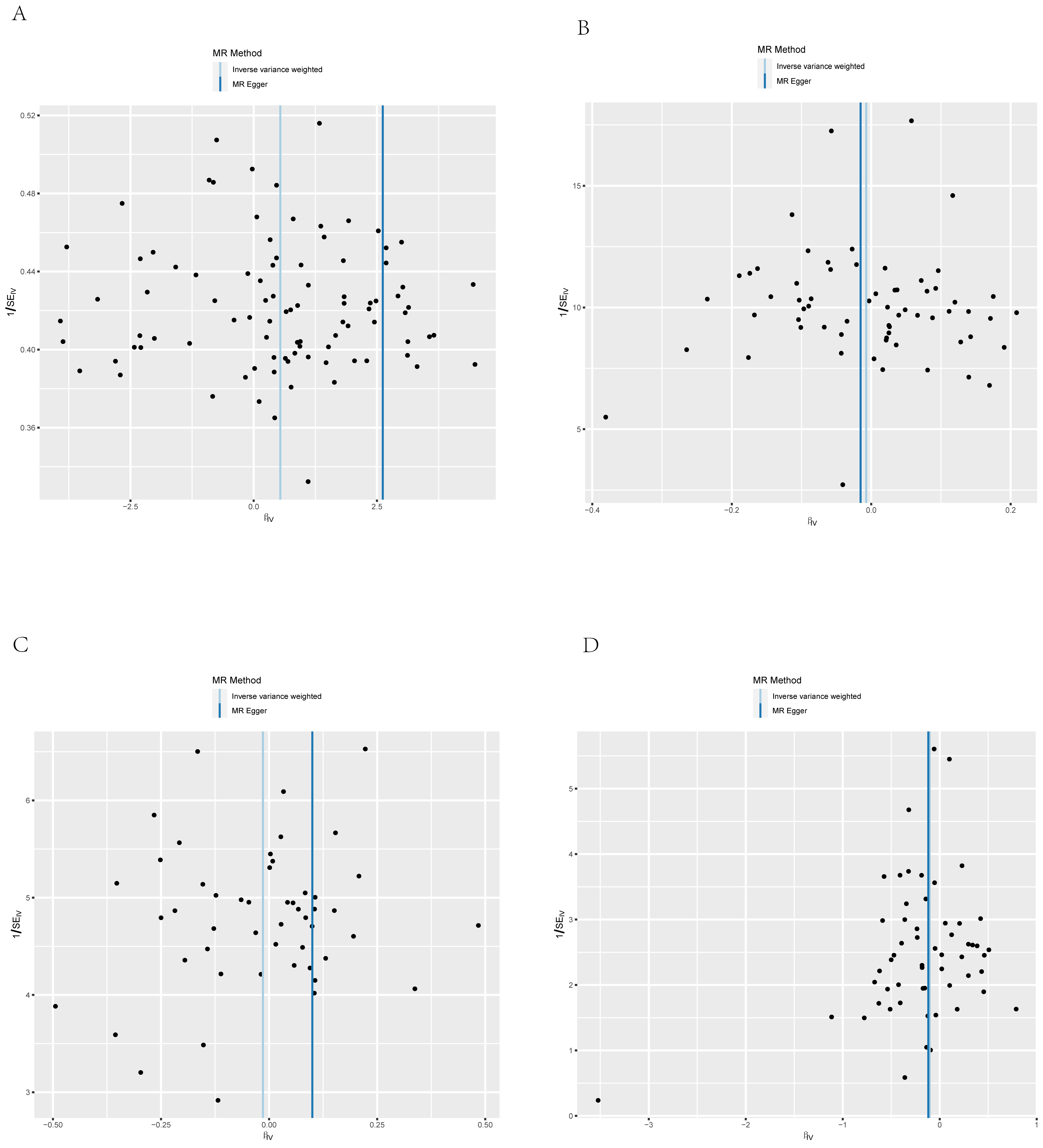

Supplement: Supplementary Figure 7 — Funnel plot of the causal association between Lifestyle Factors in Eastern Asia and GC. (A) Smoking and GC; (B) Alcohol intake and GC; (C) Coffee intake and GC; (D) Tea intake and GC. GC, Gastric cancer. [file Image_7.tif]

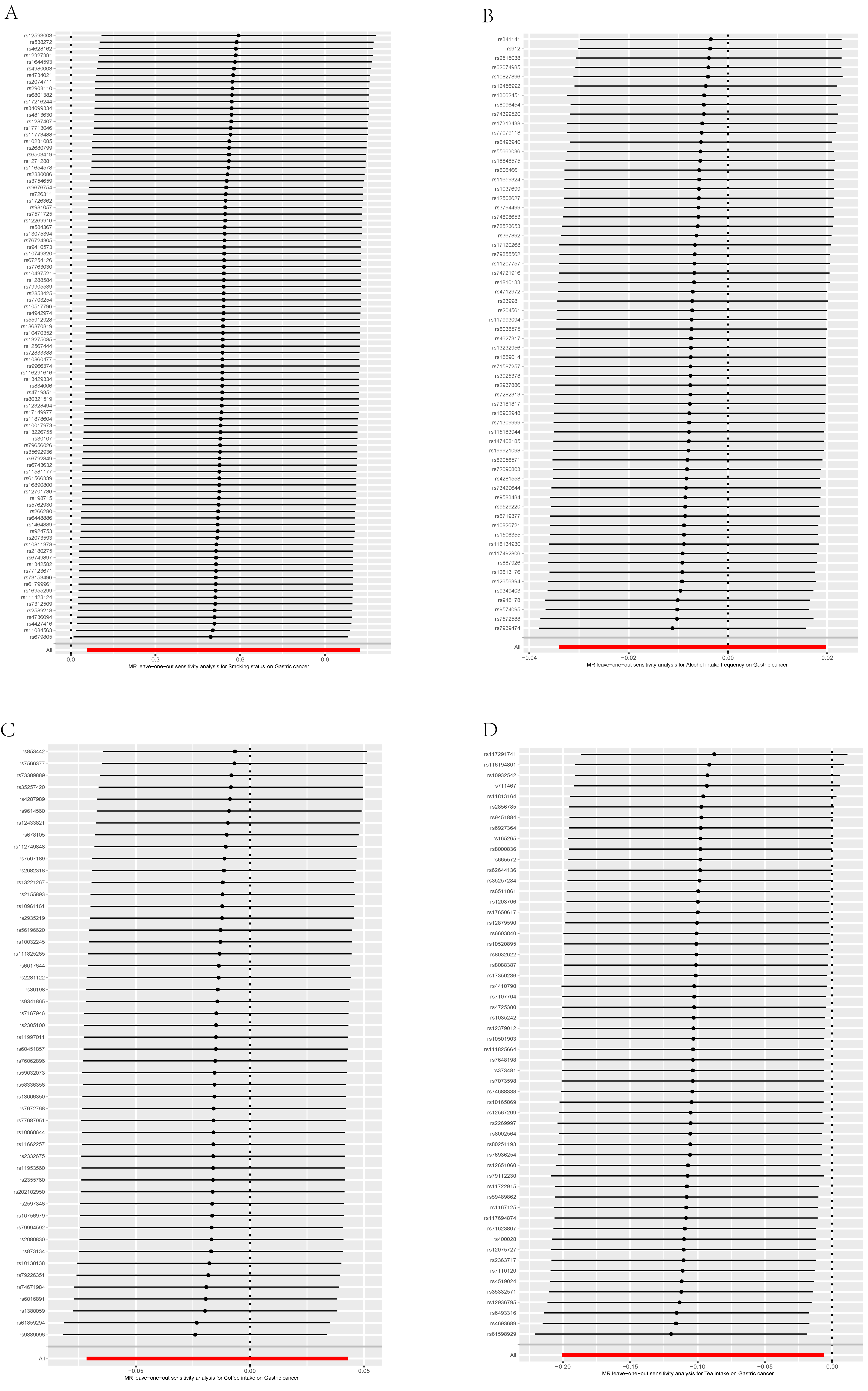

Supplement: Supplementary Figure 8 — Leave-one-out test plot of the causal association between Lifestyle Factors in Eastern Asia and GC. (A) Smoking and GC; (B) Alcohol intake and GC; (C) Coffee intake and GC; (D) Tea intake and GC. GC, Gastric cancer. [file Image_8.tif]
